# Colorectal Cancer Screening Based on Fecal Immunochemical Test and Risk Assessment: A Population-based Study Including Two Million Participants in China

**DOI:** 10.2188/jea.JE20240252

**Published:** 2025-06-05

**Authors:** Dong Hang, Chen Zhu, Xiaolin Yang, Jinjin He, Huizhang Li, Tingting Pan, Le Wang, Shi Wang, Wei Wu, Jieming Zhong, Weiwei Gong, Meng Zhu, Ci Song, Hongxia Ma, Ni Li, Yanfei Qiu, Guangfu Jin, Zhibin Hu, Lingbin Du, Xiangdong Cheng, Hongbing Shen

**Affiliations:** 1 Department of Epidemiology, Jiangsu Key Lab of Cancer Biomarkers, Prevention and Treatment, Collaborative Innovation Center for Cancer Personalized Medicine, School of Public Health, Nanjing Medical University, Nanjing, China; 2 Department of Cancer Prevention, Zhejiang Cancer Hospital, Hangzhou Institute of Medicine (HIM), Chinese Academy of Sciences, Hangzhou, China; 3 Department of Endoscopy, Zhejiang Cancer Hospital, Hangzhou Institute of Medicine (HIM), Chinese Academy of Sciences, Hangzhou, China; 4 Department of Pathology, Zhejiang Cancer Hospital, Hangzhou Institute of Medicine (HIM), Chinese Academy of Sciences, Hangzhou, China; 5 Department of Chronic and Noncommunicable Disease Control and Prevention, Zhejiang Provincial Center for Disease Control and Prevention, Hangzhou, China; 6 Office of Cancer Screening, National Cancer Center/National Clinical Research Center for Cancer/Cancer Hospital, Chinese Academy of Medical Sciences and Peking Union Medical College, Beijing, China; 7 Department of Gastric Surgery, Zhejiang Cancer Hospital, Hangzhou Institute of Medicine (HIM), Chinese Academy of Sciences, Hangzhou, China

**Keywords:** colonoscopy, colorectal cancer, screening, fecal immunochemical test, risk assessment

## Abstract

**Background:**

The fecal immunochemical test (FIT) has been widely used in colorectal cancer (CRC) screening, yet the practical performance of FIT combined with questionnaire-based risk assessment (QRA) remains undetermined. Moreover, risk factors for distinct CRC precursors identified in screening have been rarely compared.

**Methods:**

This study was based on a population-based CRC screening in China, with 2,120,340 participants completing both FIT and QRA. Those with positive FIT or high QRA scores were recommended for colonoscopy. We reported the compliance, detection rate, and colonoscopy workload according to FIT and QRA results. We also explored risk factors for conventional adenomas and serrated polyps.

**Results:**

The compliance rate of colonoscopy in the subgroup of FIT (+) and QRA (+) was 41.4%, higher than the rates in FIT (+) and QRA (−), as well as FIT (−) and QRA (+), which were 38.7% (*P* < 0.001) and 16.4% (*P* < 0.001), respectively. The corresponding detection rates of advanced neoplasia were 18.2%, 13.2%, and 9.3% (all *P* < 0.001), respectively. Moreover, the required numbers of colonoscopies to detect one advanced neoplasia in the three subgroups were 5.5, 7.6, and 10.8, respectively. Increased body mass index, smoking, alcohol consumption, red meat intake, and type 2 diabetes were associated with higher risk of advanced adenomas and advanced serrated polyps, whereas vegetable intake was inversely associated with advanced adenomas.

**Conclusion:**

FIT and QRA can synergistically identify individuals at high risk of colorectal advanced neoplasia, with those testing positive for both deserving immediate attention. Modifiable factors were identified to complement screening for preventing CRC precursors.

## INTRODUCTION

Colorectal cancer (CRC) has emerged as the third most common malignancy and the second leading cause of cancer death worldwide.^1^ Although randomized controlled trials have demonstrated the effectiveness of CRC screening in reducing CRC mortality, the low participation rate and diagnostic yield of colonoscopy in the general population remain a major challenge in many countries.^2^^,^^3^

Pre-selection of individuals who are most likely to benefit from screening has great potential to improve the cost-effectiveness of screening programs.^4^^,^^5^ In such pre-selection, the fecal immunochemical test (FIT) is most often used. Although FIT is safe, inexpensive, and easy to perform, its low sensitivity for detecting advanced adenomas limits the potential to further reduce CRC incidence.^6^^,^^7^ Notably, several studies have suggested that a combined use of FIT and CRC risk factors derived from questionnaires may improve the sensitivity for detecting colorectal advanced neoplasia.^5^^,^^8^^,^^9^ Nevertheless, current evidence is sparse regarding the performance of such combined strategy in a real-world screening setting.

Therefore, we leveraged up-to-date data from a large-scale CRC screening program conducted in China and evaluated the compliance, detection rate, and resource load of colonoscopies in different subgroups of FIT and questionnaire-based risk assessment (QRA) results. We also explored and compared risk factors for distinct CRC precursors, which would together provide new insights for developing more effective colonoscopy screening and prevention strategies.

## METHODS

### Study design

The current study focused on one-time screening of CRC. Participants were derived from an ongoing demonstration project of community-based CRC screening launched in June 2020. We invited individuals aged 50 to 74 years, residing in 11 cities within Zhejiang Province, China, and who had registered their residence with the local administrative office to participate in the study. To encourage participation, individuals aged 40–49 years were not turned away. Exclusion criteria included a previous history of CRC, colon resection, receipt of cancer therapy, and health problems that were not suitable for colonoscopy. Participants were first asked to undertake FIT and QRA by trained staff, and those who were FIT-positive or considered at high risk via QRA were then recommended for a diagnostic colonoscopy at designated hospitals. The program was approved by the Ethics Committee and Institutional Review Board of Zhejiang Cancer Hospital (IRB-2023-464), and all participants provided written informed consent.

### FIT procedure

Fecal occult blood was detected via qualitative FIT using immunogold labeling dipsticks.^10^ Healthcare providers distributed two collection kits to each participant and instructed them to collect 10–50 mg stool at home twice in 2 consecutive weeks. For participants with either one or both of the two tests positive, they were scheduled for subsequent diagnostic colonoscopy.

### Risk assessment

Demographic information, lifestyle factors, and medical history were collected through in-person interviews using a structured questionnaire. Participants were considered at high risk if they had one of the following conditions: (a) a history of colorectal polyps; (b) a family history of familial adenomatous polyposis in a first-degree relative; or (c) a modified Asia-Pacific Colorectal Screening (APCS) score ≥5 points. The APCS score, based on age, sex, family history of CRC, and smoking, was developed and validated for predicting the risk of advanced neoplasia in asymptomatic participants from 11 countries in Asia.^11^ The modified APCS score was calculated based on age (<54 years, 0 point; 55–64 years, 1 point; 65–74 years, 2 points), sex (female, 0 point; male, 1 point), family history of CRC among first-degree relatives (no, 0 point; yes, 1 point), smoking status (never smokers, 0 point; current or past smokers, 1 point), and BMI (<23 kg/m^2^, 0 point; ≥23 kg/m^2^, 1 point).^12^

### Colonoscopy diagnosis

Colonoscopies were performed in designated hospitals by experienced endoscopists following a standard procedure. Advanced adenomas and advanced serrated polyps were regarded as CRC precursors. Colorectal advanced neoplasia included CRC, advanced adenomas, and advanced serrated polyps. The definitions of advanced adenomas, advanced serrated polyps, and advanced colorectal polyps were shown in the table footnotes.

### Statistical analysis

Standardized mean differences (SMDs) were used to compare the distribution of characteristics between the attendants and non-attendants of colonoscopic screening.^13^ The colonoscopy detection rates of classified colorectal lesions were calculated according to different subgroups. The number of colonoscopies needed to detect one colonic lesion was estimated to assess the resource load. We also evaluated risk factors in relation to advanced adenomas and advanced serrated polyps separately using unconditional logistic regression models, and compared the associations between the two lesions through case-only analyses.^14^^,^^15^ The missing rates of covariates were very small, ranging from 0.0% to 3.29%. We did not impute the missing values because each covariate was treated as an exposure. The data of participants with missing values of covariates were automatically neglected in the regression models. The risk assessment accuracy of the new risk score, which incorporated factors related to CRC precursors into the modified APCS score, was assessed by the area under the receiver operating characteristic curve (AUC). All statistical analyses were performed using R (version 4.1.0; R Foundation for Statistical Computing, Vienna, Austria) statistical software. Tests were two-sided, and *P* values <0.05 were considered statistically significant.

## RESULTS

### Participation rate and compliance of colonoscopy

During the period from June to December 2020, a total of 3,976,152 eligible individuals were invited, among whom 2,120,340 (53.3%) completed both FIT and QRA. Of 409,293 (19.3%) participants with positive FIT results or high QRA scores, 123,414 underwent colonoscopy examination, with an overall compliance rate of 30.2%. After excluding participants with incomplete pathologic data or unclassified lesions (*n* = 406), 123,008 participants were included in the analysis. There were no significant differences in the distribution of characteristics between attendants and non-attendants of colonoscopic screening (eTable 1). According to FIT and QRA results, the compliance rate of colonoscopy for FIT (+) and QRA (+), FIT (+) and QRA (−), as well as FIT (−) and QRA (+) was 41.4%, 38.7%, and 16.4%, respectively (Table 1).

**Table 1.** Characteristics of participants and colonoscopy compliance according to FIT and QRA results

	All participants (*n* = 409,293)		FIT (+) and QRA (+) (*n* = 27,623)		FIT (+) and QRA (−) (*n* = 221,387)		FIT (−) and QRA (+) (*n* = 160,203)	
			
	Completed colonoscopy	Compliance rate (%)	Completed colonoscopy	Compliance rate (%)	Completed colonoscopy	Compliance rate (%)	Completed colonoscopy	Compliance rate (%)
Total	123,414	30.2	11,438	41.4	85,635	38.7	26,341	16.4
Sex, *n* (%)								
Female	56,882 (46.1)	33.1	2,713 (23.7)	41.4	46,842 (54.7)	37.2	7,327 (27.8)	18.5
Male	66,532 (53.9)	28.0	8,725 (76.3)	41.4	38,793 (45.3)	40.6	19,014 (72.2)	15.8
Age, *n* (%)								
40–49 years	3,527 (2.9)	46.2	153 (1.3)	50.8	2,981 (3.5)	50.8	393 (1.5)	26.8
50–59 years	40,057 (32.5)	37.7	2,074 (18.1)	47.6	32,688 (38.2)	42.3	5,295 (20.1)	21.4
60–69 years	59,790 (48.4)	28.2	6,472 (56.6)	41.3	38,639 (45.1)	37.3	14,679 (55.7)	15.9
70–74 years	20,040 (16.2)	24.0	2,739 (23.9)	37.6	11,327 (13.2)	32.5	5,974 (22.7)	14.4
BMI, *n* (%)								
18.5 < BMI < 24 kg/m^2^	64,190 (53.9)	32.5	4,720 (42.2)	42.8	48,550 (59.0)	39.4	10,920 (42.4)	17.2
24 ≤ BMI < 28 kg/m^2^	45,371 (38.1)	27.9	5,380 (48.1)	40.6	27,649 (33.6)	38.5	12,342 (48.0)	15.9
BMI ≥ 28 kg/m^2^	9,611 (8.1)	27.6	1,075 (9.6)	39.7	6,061 (7.4)	35.9	2,475 (9.6)	16.2
Smoking, *n* (%)								
Non-current	95,336 (77.3)	32.7	6,509 (56.9)	42.2	72,842 (85.1)	38.7	15,985 (60.7)	18.2
Current, <30 pack years	11,509 (9.3)	24.2	1,954 (17.1)	40.9	5,261 (6.1)	39.6	4,294 (16.3)	14.5
Current, ≥30 pack years	16,525 (13.4)	23.5	2,970 (26.0)	40.0	7,506 (8.8)	37.6	6,049 (23.0)	14.1
Alcohol consumption, *n* (%)								
<5 g/day	88,770 (73.8)	31.7	6,393 (58.1)	41.5	66,494 (79.3)	38.9	15,883 (62.4)	17.0
5–14.9 g/day	6,590 (5.5)	27.5	869 (7.9)	42.2	3,785 (4.5)	38.3	1,936 (7.6)	16.1
15–29.9 g/day	7,553 (6.3)	27.0	1,014 (9.2)	41.5	4,349 (5.2)	38.3	2,190 (8.6)	15.4
≥30 g/day	17,424 (14.5)	26.3	2,725 (24.8)	40.9	9,269 (11.0)	37.5	5,430 (21.3)	15.5
Vegetable intake, *n* (%)								
≤3 days/week	10,105 (8.2)	33.8	831 (7.3)	43.0	7,414 (8.7)	42.6	1,860 (7.1)	17.6
>3 days/week	113,309 (91.8)	29.9	10,607 (92.7)	41.3	78,221 (91.3)	38.3	24,481 (92.9)	16.4
Fruit intake, *n* (%)								
≤3 days/week	65,363 (53.0)	29.8	6,241 (54.6)	40.9	44,993 (52.5)	37.3	14,129 (53.6)	16.9
>3 days/week	58,051 (47.0)	30.6	5,197 (45.4)	42.0	40,642 (47.5)	40.4	12,212 (46.4)	15.9
Red meat intake, *n* (%)								
≤3 days/week	79,378 (64.3)	29.8	7,130 (62.3)	40.8	56,000 (65.4)	37.7	16,248 (61.7)	16.2
>3 days/week	44,036 (35.7)	30.8	4,308 (37.7)	42.4	29,635 (34.6)	40.8	10,093 (38.3)	16.8
Cereal intake, *n* (%)								
≤3 days/week	89,095 (72.2)	30.4	8,359 (73.1)	41.9	61,979 (72.4)	39.0	18,757 (71.2)	16.5
>3 days/week	34,319 (27.8)	29.5	3,079 (26.9)	40.1	23,656 (27.6)	37.9	7,584 (28.8)	16.4
Prevalent dyslipidemia, *n* (%)								
No	114,593 (92.9)	30.3	10,300 (90.1)	41.5	80,677 (94.2)	38.8	23,616 (89.7)	16.3
Yes	8,821 (7.1)	28.1	1,138 (9.9)	40.2	4,958 (5.8)	36.8	2,725 (10.3)	18.0
Type 2 diabetes, *n* (%)								
No	112,252 (91.0)	30.6	10,152 (88.8)	41.8	78,813 (92.0)	39.1	23,287 (88.4)	16.6
Yes	11,162 (9.0)	26.0	1,286 (11.2)	38.3	6,822 (8.0)	34.4	3,054 (11.6)	15.5
Family history of CRC, *n* (%)								
No	115,344 (96.1)	30.2	10,149 (91.4)	40.9	81,545 (98.0)	38.6	23,650 (92.2)	16.2
Yes	4,650 (3.9)	29.1	960 (8.6)	46.4	1,685 (2.0)	43.7	2,005 (7.8)	20.0

BMI, body mass index, CRC, colorectal cancer, FIT, fecal immunochemical test, QRA, questionnaire-based risk assessment.

### Detection rate of colorectal lesions

**Figure 1.**
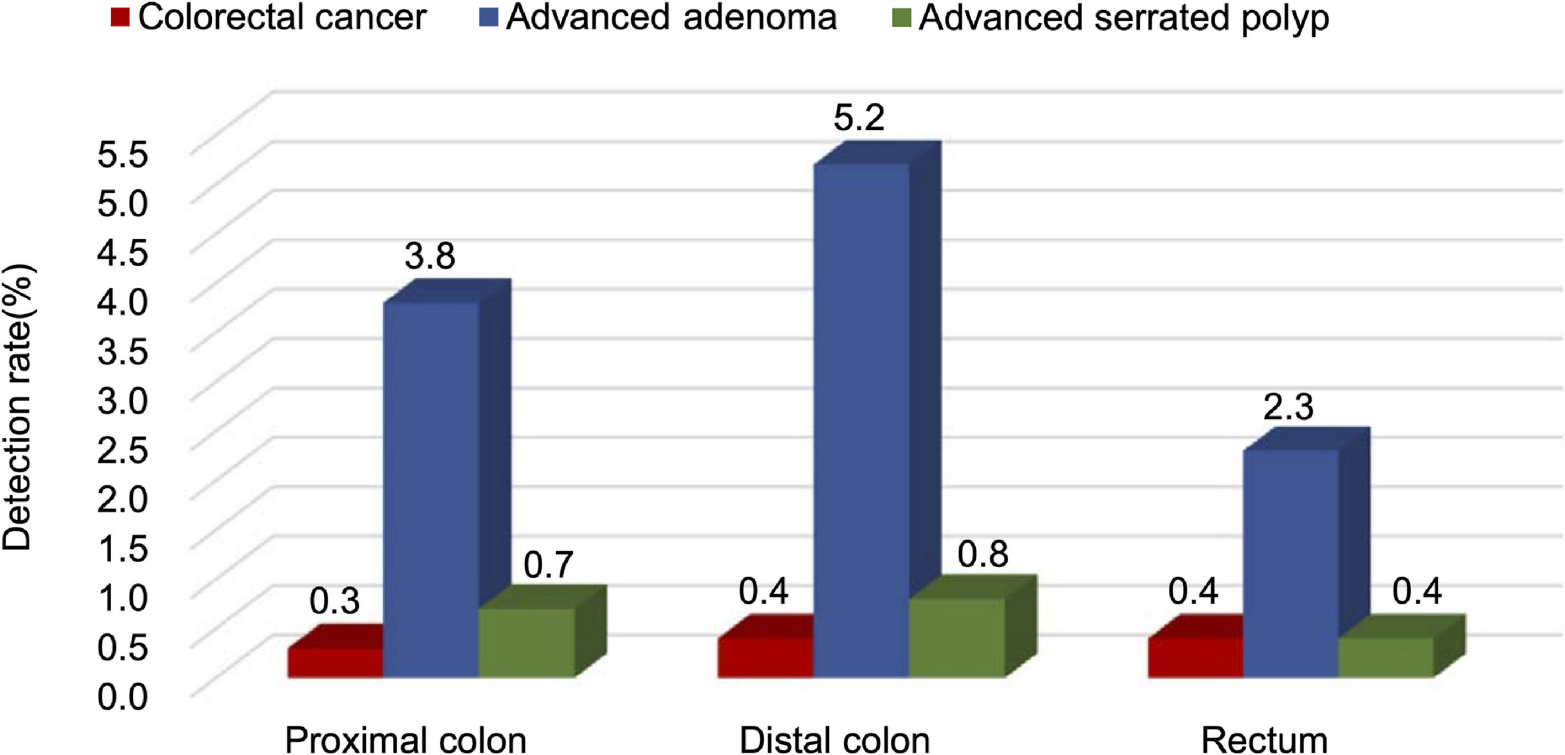
Detection rates of colorectal cancer and advanced lesions according to anatomic subsite. Proximal colon group included terminal ileum, cecum, ascending colon, hepatic flexure, transverse colon, and splenic flexure. Distal colon group included descending colon and sigmoid colon. Rectum group included rectum and anus

Among 123,008 participants who had confirmed pathological results, the detection rates of CRC, advanced adenomas, and advanced serrated polyps were 1.1% (95% confidence interval [CI], 1.0–1.1%), 10.3% (95% CI, 10.1–10.4%), and 1.8% (95% CI, 1.7–1.9%), respectively, yielding a detection rate of 12.8% for colorectal advanced neoplasia (Table 2). The detection rates of CRC in 11 cities ranged from 0.4% to 0.8%, with a median rate of 0.6% (eTable 2). The detection rates according to anatomic sites are displayed in Figure 1. We observed that the detection rate of CRC was similar across the subsite (ranging from 0.3% to 0.4%), while the rates of advanced adenomas and advanced serrated polyps were highest in the distal colon (5.2% and 0.8%, respectively). Among those with benign lesions or normal status during screening, the cancer registry identified 191 CRC cases within 6 months after colonoscopy screening and 106 CRC cases between 6 and 36 months later (eTable 3). Most of the CRC patients diagnosed within 6 months had contraindications, such as the use of anticoagulants and coagulation disorders, which made them ineligible for biopsy; thus, the endoscopist deemed these cases as benign lesions and recommended a repeat colonoscopy once the contraindications disappeared. Because these individuals chose to undergo a repeated colonoscopy in undesignated hospitals, we did not receive their pathological records but found these cases in the cancer registry.

**Table 2.** Colonoscopy detection rates of colorectal lesions and number of colonoscopies to detect one colorectal lesion according to FIT and QRA results

Outcome	All participants (*n* = 123,008)			FIT (+) and QRA (+) (*n* = 11,354)			FIT (+) and QRA (−) (*n* = 85,394)			FIT (−) and QRA (+) (*n* = 26,260)		
			
	*N*	Detection rate (95% CI), %	Resource load^e^, *N* (95% CI)	*N*	Detection rate (95% CI), %	Resource load^e^, *N* (95% CI)	*N*	Detection rate (95% CI), %	Resource load^e^, *N* (95% CI)	*N*	Detection rate (95% CI), %	Resource load^e^, *N* (95% CI)
CRC	1,324	1.1 (1.0–1.1)	92.9 (88.2–98.2)	174	1.5 (1.3–1.8)	65.3 (56.9–76.5)	1,088	1.3 (1.2–1.3)	78.5 (74.1–83.4)	62	0.2 (0.2–0.3)	423.5 (339.2–563.7)
Advanced neoplasia^a^	15,756	12.8 (12.6–13.0)	7.8 (7.7–7.9)	2,062	18.2 (17.5–18.9)	5.5 (5.3–5.7)	11,260	13.2 (13.0–13.4)	7.6 (7.5–7.7)	2,434	9.3 (8.9–9.6)	10.8 (10.4–11.2)
Advanced colorectal polyps	14,432	11.7 (11.6–11.9)	8.5 (8.4–8.7)	1,888	16.6 (15.9–17.3)	6.0 (5.8–6.3)	10,172	11.9 (11.7–12.1)	8.4 (8.2–8.6)	2,372	9.0 (8.7–9.4)	11.1 (10.7–11.5)
Advanced adenoma^b^	12,610	10.3 (10.1–10.4)	9.8 (9.6–9.9)	1,667	14.7 (14.0–15.3)	6.8 (6.5–7.1)	9,009	10.5 (10.3–10.8)	9.5 (9.3–9.7)	1,934	7.4 (7.0–7.7)	13.6 (13.0–14.2)
Advanced serrated polyp^c^	2,222	1.8 (1.7–1.9)	55.4 (53.2–57.7)	293	2.6 (2.3–2.9)	38.8 (34.8–43.7)	1,429	1.7 (1.6–1.8)	59.8 (56.8–63.0)	500	1.9 (1.7–2.1)	52.5 (48.3–57.5)
Non-advanced colorectal polyps	25,461	20.7 (20.5–20.9)	4.8 (4.8–4.9)	2,795	24.6 (23.8–25.4)	4.1 (3.9–4.2)	15,962	18.7 (18.4–19.0)	5.3 (5.3–5.4)	6,704	25.5 (25.0–26.1)	3.9 (3.8–4.0)
Non-advanced adenoma	21,634	17.6 (17.4–17.8)	5.7 (5.6–5.8)	2,391	21.1 (20.3–21.8)	4.7 (4.6–4.9)	13,653	16.0 (15.7–16.2)	6.3 (6.2–6.4)	5,590	21.3 (20.8–21.8)	4.7 (4.6–4.8)
Non-advanced serrated polyp	4,822	3.9 (3.8–4.0)	25.5 (24.8–26.2)	528	4.7 (4.3–5.0)	21.5 (19.9–23.5)	2,893	3.4 (3.3–3.5)	29.5 (28.5–30.6)	1,401	5.3 (5.1–5.6)	18.7 (17.8–19.7)
Other benign lesions^d^	26,999	21.9 (21.7–22.2)		2,741	24.1 (23.4–24.9)		17,916	21.0 (20.7–21.3)		6,342	24.2 (23.6–24.7)	
Normal	54,792	44.5 (44.3–44.8)		3,756	33.1 (32.2–33.9)		40,256	47.1 (46.8–47.5)		10,780	41.1 (40.5–41.6)	

CI, confidence interval; CRC, colorectal cancer; FIT, fecal immunochemical test; QRA, questionnaire-based risk assessment.

^a^Advanced neoplasia included CRC and advanced colorectal polyps.

^b^Advanced adenomas were defined as adenomas with any of the following features: ≥10 mm in size, tubulovillous or villous histology, or high-grade dysplasia.

^c^Advanced serrated polyps were defined as serrated polyps of either ≥10 mm in size or containing dysplasia.

^d^Other benign lesions included chronic colitis, chronic proctitis, non-adenomatous polyps, and low-grade dysplasia.

^e^Number of colonoscopies to detect one colorectal lesion.

In the three subgroups of FIT (+) and QRA (+), FIT (+) and QRA (−), as well as FIT (−) and QRA (+), the corresponding detection rates of CRC were 1.5% (95% CI, 1.3–1.8%), 1.3% (95% CI, 1.2–1.3%), and 0.2% (95% CI, 0.2–0.3%), respectively; the rates of advanced adenomas were 14.7% (95% CI, 14.0–15.3%), 10.5% (95% CI, 10.3–10.8%), and 7.4% (95% CI, 7.0–7.7%), respectively; and the rates of advanced serrated polyps were 2.6% (95% CI, 2.3–2.9%), 1.7% (95% CI, 1.6–1.8%), and 1.9% (95% CI, 1.7–2.1%), respectively, yielding the detection rates of 18.2%, 13.2%, and 9.3%, respectively, for colorectal advanced neoplasia (Table 2). Significant differences in the detection rates of advanced adenomas, advanced serrated polyps, and advanced neoplasia were observed between the subgroups (eTable 4). Among the 1,711,047 participants with FIT (−) and QRA (−), the CRC incidence rate was 0.14% according to the cancer registry.

### Resource load of colonoscopy

The required number of colonoscopies for detection of one CRC, one advanced adenoma, and one advanced serrated polyp was 92.9 (95% CI, 88.2–98.2), 9.8 (95% CI, 9.6–9.9), and 55.4 (95% CI, 53.2–57.7), respectively. Concerning the FIT and QRA results, the required number of colonoscopies to detect one CRC was 65.3 (95% CI, 56.9–76.5) for FIT (+) and QRA (+), 78.5 (95% CI, 74.1–83.4) for FIT (+) and QRA (−), and 423.5 (95% CI, 339.2–563.7) for FIT (−) and QRA (+); the corresponding number to detect one advanced adenoma was 6.8 (95% CI, 6.5–7.1), 9.5 (95% CI, 9.3–9.7), and 13.6 (95% CI, 13.0–14.2), respectively; and the corresponding number to detect one advanced serrated polyp was 38.8 (95% CI, 34.8–43.7), 59.8 (95% CI, 56.8–63.0), and 52.5 (95% CI, 48.3–57.5), respectively (Table 2).

### Risk factors of advanced colorectal polyps

Male gender, older age, higher BMI, current smoking, alcohol consumption, red meat intake, and type 2 diabetes were associated with higher risks of advanced adenomas and advanced serrated polyps (all *P* values <0.05) (eTable 5). The associations with male gender, older age, and alcohol consumption tended to be stronger for advanced adenomas than advanced serrated polyps, whereas the association with smoking was stronger for advanced serrated polyps (*P* for heterogeneity <0.05). In addition, a more frequent intake of vegetables was associated with a lower risk of advanced adenomas (OR 0.91; 95% CI, 0.84–0.98) but not advanced serrated polyps. The new risk score had an AUC of 0.65 (95% CI, 0.64–0.65), which was higher than that of the modified APCS score (0.63; 95% CI, 0.63–0.63) (eFigure 1).

## DISCUSSION

Although colonoscopy is regarded as the gold standard tool of CRC screening, the compliance and efficiency of colonoscopy in the general population are not optimal. Pre-selection of high-risk individuals for CRC screening has been suggested as a potential cost-effective approach. In a previous study involving 1,381,561 participants in China, 182,927 (13.2%) were evaluated to be high risk for CRC by a risk score system named the Harvard Risk Index, yielding the colonoscopy compliance of 14.0%.^3^ The rate was close to that of the FIT (−) and QRA (+) subgroup in our study (16.4%), indicating that relying solely on questionnaire-based risk stratification is far from satisfactory in a real-world setting. Our study showed that the FIT (+) and QRA (+) subgroup had the highest compliance of 41.4%, indicating the usefulness of the strategy combing FIT and QRA to enhance the colonoscopy acceptance. QRA has the potential to enable participants to better understand their individual risk of CRC and therefore to appreciate the need for colonoscopy.

In the current study, overall detection rates of CRC and advanced polyps were 1.1% and 11.7%, respectively. The results are in line with those from a Thailand study combining risk assessment and FIT to prioritize colonoscopy in asymptomatic participants, which reported a detection rate of 0.7% for CRC and 11.2% for advanced adenomas.^5^ Furthermore, the Thailand study and ours showed that the FIT (+) and QRA (+) subgroup had higher detection rates of CRC and advanced polyps compared with the other subgroups, indicating that individuals with positive FIT and QRA results should be given priority for colonoscopy because they are most likely to have advanced neoplasia.

We also found that the number of colonoscopies needed for detection of one advanced neoplasia was lowest in those with positive FIT results and high QRA scores, suggesting the potential of the combined strategy to reduce colonoscopy workload. Moreover, leveraging data from a CRC screening trial in the Netherlands, Stegeman et al developed a model combining FIT with age, calcium intake, and family history of CRC, which showed a c-statistic of 0.76 compared to 0.69 with FIT only for advanced neoplasia.^9^ The findings and ours together support that the screening performance for advanced neoplasia could be improved by using FIT in combination with risk factors compared with using FIT alone.

We noticed a variation in colonoscopy compliance rates and detection rates of advanced colorectal polyps across different cities. The causes for the discrepancies are complex. Socioeconomic status, education background, family history of CRC, and history of colonic polyps may contribute to the variation in compliance rates, while obesity, dietary habits, lifestyle factors, and family history of CRC may contribute to the variation in detection rates.^3^^,^^16^ Meanwhile, a total of 106 interval cancer cases were identified by the cancer registry between 6 and 36 months after colonoscopy screening, which may be attributed to missed lesions, unresected or incompletely resected lesions, or new rapid-growth cancer.^17^

Conventional adenomas and serrated polyps are precursor lesions for CRC but derive from distinct biological pathways.^18^^,^^19^ Although conventional adenomas and serrated polyps share some CRC risk factors, we found that current smoking was more strongly associated with advanced serrated polyps than advanced conventional polyps, whereas alcohol consumption was more strongly associated with advanced adenomas. In line with our findings, previous studies also observed that the association with smoking was stronger for serrated polyps than conventional adenomas.^20^^,^^21^ For alcohol consumption, in support of our results, a prior meta-analysis reported a dose-response association between alcohol intake and risk of conventional adenomas,^22^ and another meta-analysis found a positive association between alcohol consumption and serrated polyps.^23^ Additionally, we found that more frequent intake of vegetables was associated with a lower risk of advanced adenomas, consistent with the results from several previous studies.^24^^,^^25^ However, no statistically significant association was observed between vegetable intake and advanced serrated polyps. In support of our findings, several large-scale cohort studies reported no significant association between fiber intake, a key antineoplastic component in vegetables, and serrated polyps.^26^^,^^27^ We could not exclude the influence of unknown confounders on the estimation of the association between vegetable intake and advanced serrated polyps. Therefore, future investigations are warranted to clarify the association between vegetable consumption and serrated polyps.

Several limitations need to be noted when interpreting our results. First, the information on lifestyle, diet, and medical history was self-reported and thus might be subject to measurement errors. Second, the compliance rate of colonoscopy for high-risk participants remained suboptimal, which may underestimate the detection rates of the combined strategy. Third, it should be noted that a very small proportion of participants who had contraindications to biopsy (191/123,008; 0.16%) in the screening were identified having CRC through cancer registration within 6 months, which might dilute screening effectiveness. To address this, we would consider these cases as CRC, but not benign lesions, in future assessment of the effectiveness of colonoscopy. Detailed sensitivity analyses (eg, including and excluding these cases) would be also performed to confirm the robustness of findings. Finally, the current study is unable to evaluate the impact of screening on CRC mortality. Our future endeavors will focus on conducting long-term follow-up to address this critical aspect.

In conclusion, the combination of FIT and QRA can enhance the efficiency of CRC screening by identifying individuals at high risk of colorectal advanced neoplasia and reducing colonoscopy workload. Moreover, we identified several modified factors for distinct CRC precursors, which represent a complement to colonoscopy screening for reducing CRC incidence.

## SUPPLEMENTARY MATERIAL

eTable 1. The characteristics of the attendants and non-attendants of colonoscopy among the participants with positive FIT results or high QRA scores

eTable 2. The age-standardized colonoscopy compliance rates and age-standardized detection rates of colorectal lesions in different cities

eTable 3. CRC incidence among the participants with benign lesions or normal status during 2020–2023

eTable 4. The statistical tests for detection rates according to FIT and QRA results

eTable 5. Associations of risk factors with advanced adenomas and advanced serrated polyps

eFigure 1. Receiver operating characteristic curves for colorectal cancer based on the new risk score, Asia-Pacific Colorectal Screening (APCS) score and the modified APCS score

## References

[1] Sung H, Ferlay J, Siegel RL, . Global cancer statistics 2020: GLOBOCAN estimates of incidence and mortality worldwide for 36 cancers in 185 countries. CA Cancer J Clin. 2021;71:209–249. 10.3322/caac.21660 33538338

[2] Kaminski MF, Robertson DJ, Senore C, Rex DK. Optimizing the quality of colorectal cancer screening worldwide. Gastroenterology. 2020;158:404–417. 10.1053/j.gastro.2019.11.026 31759062

[3] Chen H, Li N, Ren J, ; group of Cancer Screening Program in Urban China (CanSPUC). Participation and yield of a population-based colorectal cancer screening programme in China. Gut. 2019;68:1450–1457. 10.1136/gutjnl-2018-317124 30377193

[4] Zhong GC, Sun WP, Wan L, Hu JJ, Hao FB. Efficacy and cost-effectiveness of fecal immunochemical test versus colonoscopy in colorectal cancer screening: a systematic review and meta-analysis. Gastrointest Endosc. 2020;91:684–697.e15. 10.1016/j.gie.2019.11.035 31790657

[5] Aniwan S, Rerknimitr R, Kongkam P, . A combination of clinical risk stratification and fecal immunochemical test results to prioritize colonoscopy screening in asymptomatic participants. Gastrointest Endosc. 2015;81:719–727. 10.1016/j.gie.2014.11.035 25708760

[6] Helsingen LM, Vandvik PO, Jodal HC, . Colorectal cancer screening with faecal immunochemical testing, sigmoidoscopy or colonoscopy: a clinical practice guideline. BMJ. 2019;367:l5515. 10.1136/bmj.l5515 31578196

[7] Buskermolen M, Cenin DR, Helsingen LM, . Colorectal cancer screening with faecal immunochemical testing, sigmoidoscopy or colonoscopy: a microsimulation modelling study. BMJ. 2019;367:l5383. 10.1136/bmj.l5383 31578177 PMC6774435

[8] Cooper JA, Parsons N, Stinton C, . Risk-adjusted colorectal cancer screening using the FIT and routine screening data: development of a risk prediction model. Br J Cancer. 2018;118:285–293. 10.1038/bjc.2017.375 29096402 PMC5785737

[9] Stegeman I, de Wijkerslooth TR, Stoop EM, . Combining risk factors with faecal immunochemical test outcome for selecting CRC screenees for colonoscopy. Gut. 2014;63:466–471. 10.1136/gutjnl-2013-305013 23964098

[10] Gong Y, Peng P, Bao P, . The implementation and first-round results of a community-based colorectal cancer screening program in Shanghai, China. Oncologist. 2018;23:928–935. 10.1634/theoncologist.2017-0451 29540604 PMC6156172

[11] Yeoh KG, Ho KY, Chiu HM, ; Asia-Pacific Working Group on Colorectal Cancer. The Asia-Pacific Colorectal Screening score: a validated tool that stratifies risk for colorectal advanced neoplasia in asymptomatic Asian subjects. Gut. 2011;60:1236–1241. 10.1136/gut.2010.221168 21402615

[12] Sung JJY, Wong MCS, Lam TYT, . A modified colorectal screening score for prediction of advanced neoplasia: a prospective study of 5744 subjects. J Gastroenterol Hepatol. 2018;33:187–194. 10.1111/jgh.13835 28561279

[13] Austin PC, Stuart EA. Moving towards best practice when using inverse probability of treatment weighting (IPTW) using the propensity score to estimate causal treatment effects in observational studies. Stat Med. 2015;34:3661–3679. 10.1002/sim.6607 26238958 PMC4626409

[14] Wang L, Lo CH, He X, . Risk factor profiles differ for cancers of different regions of the colorectum. Gastroenterology. 2020;159:241–256.e13. 10.1053/j.gastro.2020.03.054 32247020 PMC7387153

[15] Wang M, Spiegelman D, Kuchiba A, . Statistical methods for studying disease subtype heterogeneity. Stat Med. 2016;35:782–800. 10.1002/sim.6793 26619806 PMC4728021

[16] Yu Z, Li B, Zhao S, . Uptake and detection rate of colorectal cancer screening with colonoscopy in China: a population-based, prospective cohort study. Int J Nurs Stud. 2024;153:104728. 10.1016/j.ijnurstu.2024.104728 38461798

[17] Rutter MD, Beintaris I, Valori R, . World Endoscopy Organization consensus statements on post-colonoscopy and post-imaging colorectal cancer. Gastroenterology. 2018;155:909–925.e3. 10.1053/j.gastro.2018.05.038 29958856

[18] Fearon ER, Vogelstein B. A genetic model for colorectal tumorigenesis. Cell. 1990;61:759–767. 10.1016/0092-8674(90)90186-I 2188735

[19] Snover DC. Update on the serrated pathway to colorectal carcinoma. Hum Pathol. 2011;42:1–10. 10.1016/j.humpath.2010.06.002 20869746

[20] Davenport JR, Su T, Zhao Z, . Modifiable lifestyle factors associated with risk of sessile serrated polyps, conventional adenomas and hyperplastic polyps. Gut. 2018;67:456–465. 10.1136/gutjnl-2016-312893 27852795 PMC5432410

[21] Burnett-Hartman AN, Passarelli MN, Adams SV, . Differences in epidemiologic risk factors for colorectal adenomas and serrated polyps by lesion severity and anatomical site. Am J Epidemiol. 2013;177:625–637. 10.1093/aje/kws282 23459948 PMC3657530

[22] Zhu JZ, Wang YM, Zhou QY, Zhu KF, Yu CH, Li YM. Systematic review with meta-analysis: alcohol consumption and the risk of colorectal adenoma. Aliment Pharmacol Ther. 2014;40:325–337. 10.1111/apt.12841 24943329

[23] Bailie L, Loughrey MB, Coleman HG. Lifestyle risk factors for serrated colorectal polyps: a systematic review and meta-analysis. Gastroenterology. 2017;152:92–104. 10.1053/j.gastro.2016.09.003 27639804

[24] Benito E, Cabeza E, Moreno V, Obrador A, Bosch FX. Diet and colorectal adenomas: a case-control study in Majorca. Int J Cancer. 1993;55:213–219. 10.1002/ijc.2910550208 8370618

[25] Millen AE, Subar AF, Graubard BI, ; PLCO Cancer Screening Trial Project Team. Fruit and vegetable intake and prevalence of colorectal adenoma in a cancer screening trial. Am J Clin Nutr. 2007;86:1754–1764. 10.1093/ajcn/86.6.1754 18065596

[26] He X, Wu K, Ogino S, Giovannucci EL, Chan AT, Song M. Association between risk factors for colorectal cancer and risk of serrated polyps and conventional adenomas. Gastroenterology. 2018;155:355–373.e18. 10.1053/j.gastro.2018.04.019 29702117 PMC6067965

[27] Lyu Z, Hang D, He X, . Simple prediction model for colorectal serrated polyps: development and external validation study in U.S. prospective cohorts. Cancer Prev Res (Phila). 2023;16:293–302. 10.1158/1940-6207.CAPR-22-0335 36857746

