# Supplementary material for: Colorectal Cancer Screening Based on Fecal Immunochemical Test and Risk Assessment: A Population-based Study Including Two Million Participants in China
Source: J Epidemiol. 2025 Jun 5;35(6):297–302. doi: 10.2188/jea.JE20240252 (PMC12066195; doi:10.2188/jea.JE20240252)
Supplement: Supplementary file 1 [file je-35-297-s001.pdf]

eTable 1. The characteristics of the attendants and non-attendants of colonoscopy among the participants with positive FIT results or high QRA scores

Characteristics	Attendants	Non-attendants	SMD[a]
Total	123,414	285,879	
Sex, n (%)			0.12
Female	56,882 (46.1)	115,077 (40.3)	
Male	66,532 (53.9)	170,802 (59.7)	
Age, n (%)			0.25
40–49 years	3,527 (2.9)	4,107 (1.4)	
50–59 years	40,057 (32.5)	66,247 (23.2)	
60–69 years	59,790 (48.4)	151,966 (53.2)	
70–74 years	20,040 (16.2)	63,559 (22.2)	
BMI, n (%)			0.11
18.5<BMI<24 kg/m^2	64,190 (53.9)	133,471 (48.3)	
24≤BMI<28 kg/m^2	45,371 (38.1)	117,482 (42.5)	
BMI≥28 kg/m^2	9611 (8.1)	25,244 (9.1)	
Smoking, n (%)			0.20
Non-current	95,336 (77.3)	196,004 (68.6)	
Current, <30 pack years	11,509 (9.3)	36,065 (12.6)	
Current, ≥30 pack years	16,525 (13.4)	53,691 (18.8)	
Alcohol consumption, n (%)			0.11
<5 g/day	88,770 (73.8)	191,098 (68.8)	
5–14.9 g/day	6,590 (5.5)	17,405 (6.3)	
15–29.9 g/day	7,553 (6.3)	20,427 (7.4)	
≥30 g/day	17,424 (14.5)	48,943 (17.6)	
Vegetable intake, n (%)			0.05
≤3 days/week	10,105 (8.2)	19,802 (6.9)	
>3 days/week	113,309 (91.8)	266,077 (93.1)	
Fruit intake, n (%)			0.02
≤3 days/week	65,363 (53.0)	154,281 (54.0)	
>3 days/week	58,051 (47.0)	131,598 (46.0)	
Red meat intake, n (%)			0.02
≤3 days/week	79,378 (64.3)	186,874 (65.4)	
>3 days/week	44,036 (35.7)	99,005 (34.6)	
Cereal intake, n (%)			0.02
≤3 days/week	89,095 (72.2)	203,796 (71.3)	
>3 days/week	34,319 (27.8)	82,083 (28.7)	
Prevalent dyslipidemia, n (%)			0.03
No	114,593 (92.9)	263,253 (92.1)	
Yes	8,821 (7.1)	22,626 (7.9)	
Prevalent diabetes, n (%)			0.07
No	112,252 (91.0)	254,142 (88.9)	
Yes	11,162 (9.0)	31,737 (11.1)	

Family history of CRC, n (%)			0.01
No	115,344 (96.1)	267,065 (96.0)	
Yes	4,650 (3.9)	11,305 (4.0)	

BMI, body mass index; CRC, colorectal cancer; FIT, fecal immunochemical test; QRA, questionnaire-based risk assessment; SMD, standardized mean difference.

[a] SMD >0.2 indicates a potentially relevant difference between the subgroups.

eTable 2. The age-standardized colonoscopy compliance rates and age-standardized detection rates of colorectal lesions in different cities

City	Colonoscopy compliance rates	Detection rates of colorectal lesions (95% CI)				
		CRC	Advanced neoplasia [a]	Advanced colorectal polyps [b]	Non-Advanced colorectal polyps [c]	Other benign lesions [d]
Hangzhou	34.7 (26.2–43.2)	0.7 (0.4–1.0)	9.6 (7.9–11.3)	8.9 (7.2–10.5)	14.3 (12.1–16.6)	17.9 (15.2–20.6)
Wenzhou	30.0 (26.0–34.0)	0.6 (0.2–1.0)	7.0 (5.7–8.2)	6.4 (5.2–7.6)	13.4 (8.1–18.6)	17.6 (12.6–22.6)
Jinhua	30.0 (26.4–33.6)	0.4 (0.1–0.7)	8.9 (6.2–11.6)	8.5 (5.8–11.1)	14.4 (10.7–18.0)	25.1 (19.8–30.3)
Lishui	38.5 (34.5–42.6)	0.5 (0.2–0.8)	7.5 (5.1–9.9)	7.0 (4.7–9.4)	12.6 (9.5–15.6)	30.1 (24.6–35.6)
Ningbo	39.9 (10.4–69.3)	0.4 (0.2–0.7)	11.0 (8.9–13.2)	10.6 (8.5–12.8)	18.5 (15.4–21.7)	12.0 (9.6–14.4)
Taizhou	45.9 (38.7–53.1)	0.5 (0.2–0.7)	6.3 (5.1–7.4)	5.8 (4.7–6.9)	14.4 (12.6–16.1)	28.2 (20.2–36.3)
Jiaxing	38.2 (29.0–47.3)	0.4 (0.2–0.5)	6.0 (4.9–7.0)	5.6 (4.6–6.6)	8.3 (6.2–10.5)	10.3 (7.2–13.3)
Quzhou	31.3 (23.9–38.7)	0.7 (0.2–1.3)	9.1 (4.4–13.8)	8.4 (3.8–12.9)	15.6 (10.2–21.0)	12.8 (7.6–17.9)
Shaoxing	25.5 (19.5–31.6)	0.8 (0.1–1.5)	6.9 (5.1–8.7)	6.1 (4.5–7.8)	15.8 (9.4–22.2)	19.8 (11.6–28.0)
Huzhou	25.7 (23.7–27.8)	0.8 (0.4–1.2)	9.1 (7.1–11.2)	8.3 (6.3–10.3)	21.3 (18.1–24.5)	15.5 (12.5–18.4)

Zhoushan	73.1 (35.7－100.0)	0.7 (0.2–1.3)	5.7 (3.7–7.7)	4.9 (3.1–6.8)	24.1 (0–55.6)	21.8 (17.0–26.6)

CI, confidence interval; CRC, colorectal cancer.

[a] Advanced neoplasia included CRC and advanced colorectal polyps.

[b] Advanced colorectal polyps included advanced adenoma and advanced serrated polyp. Advanced adenomas were defined as adenomas with any of the following features: ≥10 mm in size, tubulovillous or villous histology, or high-grade dysplasia. Advanced serrated polyps were defined as serrated polyps of either ≥10 mm in size or containing dysplasia.

[c] Non-Advanced colorectal polyps included non-advanced adenoma and non-advanced serrated polyp.

[d] Other benign lesions included chronic colitis, chronic proctitis, non-adenomatous polyps, and low-grade dysplasia.

eTable 3. CRC incidence among the participants with benign lesions or normal status during 2020–2023

	All participants, N (%)	FIT (+) and QRA (+), N (%)	FIT (+) and QRA (-), N (%)	FIT (-) and QRA (+), N (%)
Total	107,252	9,292	74,134	23,826
≤6 months	191 (0.18%)	22 (0.24%)	154 (0.21%)	15 (0.06%)
6 to ≤12 months	27 (0.03%)	1 (0.01%)	21 (0.03%)	5 (0.02%)
12 to ≤24 months	41 (0.04%)	6 (0.06%)	30 (0.04%)	5 (0.02%)
24 to ≤36 months	38 (0.03%)	3 (0.03%)	31 (0.04%)	4 (0.02%)

CRC, colorectal cancer; FIT, fecal immunochemical test; QRA, questionnaire-based risk assessment.

Note: The CRC cases diagnosed between 6 and 36 months after colonoscopy screening were classified as interval CRC cases.

eTable 4. The statistical tests for detection rates according to FIT and QRA results

Outcome	FIT (+) and QRA (+) *vs.* FIT (+) and QRA (-)	FIT (+) and QRA (+) *vs.* FIT (-) and QRA (+)	FIT (+) and QRA (-) *vs.* FIT (-) and QRA (+)
CRC	0.025	<0.001	<0.001
Advanced neoplasia [a]	<0.001	<0.001	<0.001
Advanced colorectal polyps	<0.001	<0.001	<0.001
Advanced adenoma [b]	<0.001	<0.001	<0.001
Advanced serrated polyp [c]	<0.001	<0.001	0.013
Non-Advanced colorectal polyps	<0.001	0.063	<0.001
Non-advanced adenoma	<0.001	0.629	<0.001
Non-advanced serrated polyp	<0.001	0.006	<0.001
Other benign lesions [d]	<0.001	0.995	<0.001
Normal	<0.001	<0.001	<0.001

CRC, colorectal cancer; FIT, fecal immunochemical test; QRA, questionnaire-based risk assessment.

Note: The Bonferroni correction was applied for multiple comparisons and *P* values<0.0125 were considered statistically significant.

[a] Advanced neoplasia included CRC and advanced colorectal polyps.

[b] Advanced adenomas were defined as adenomas with any of the following features: ≥10 mm in size, tubulovillous or villous histology, or high-grade dysplasia.

[c] Advanced serrated polyps were defined as serrated polyps of either ≥10 mm in size or containing dysplasia.

[d] Other benign lesions included chronic colitis, chronic proctitis, non-adenomatous polyps, and low-grade dysplasia.

eTable 5. Associations of risk factors with advanced adenomas and advanced serrated polyps

Factors	Advanced adenoma			Advanced serrated polyp			*P* for heterogeneity [b]
	N	Adjusted OR (95% CI) [a]	*P*	N	Adjusted OR (95% CI) [a]	*P*	
Sex							0.03
Female	3,766	1.0 (ref)		727	1.0 (ref)		
Male	7,648	1.72 (1.63–1.82)	<0.001	1,291	1.47 (1.31–1.65)	<0.001	
Age							<0.001
40–49 years	136	1.0 (ref)		37	1.0 (ref)		
50–59 years	2,718	1.99 (1.66–2.38)	<0.001	550	1.45 (1.03–2.03)	0.03	
60–69 years	6,122	3.22 (2.70–3.85)	<0.001	1,054	2.01 (1.44–2.81)	<0.001	
70–74 years	2,438	4.19 (3.49–5.02)	<0.001	377	2.35 (1.66–3.31)	<0.001	
BMI							0.13
18.5<BMI<24 kg/m^2	5,779	1.0 (ref)		981	1.0 (ref)		
24≤BMI<28 kg/m^2	4,595	1.14 (1.09–1.19)	<0.001	848	1.24 (1.13–1.36)	<0.001	
BMI≥28 kg/m^2	1,040	1.43 (1.32–1.54)	<0.001	189	1.49 (1.27–1.76)	<0.001	
Smoking							0.01
Non-current	7,775	1.0 (ref)		1,356	1.0 (ref)		
Current, <30 pack years	1,399	1.33 (1.24–1.43)	<0.001	245	1.48 (1.27–1.73)	<0.001	
Current, ≥30 pack years	2,240	1.53 (1.44–1.63)	<0.001	417	1.88 (1.65–2.14)	<0.001	
Alcohol consumption							0.01
<5 g/day	7,152	1.0 (ref)		1,317	1.0 (ref)		
5–14.9 g/day	737	1.17 (1.07–1.28)	<0.001	123	1.10 (0.90–1.33)	0.36	
15–29.9 g/day	922	1.23 (1.13–1.33)	<0.001	164	1.22 (1.02–1.45)	0.03	
⩾30 g/day	2,603	1.45 (1.37–1.54)	<0.001	414	1.27 (1.12–1.45)	<0.001	

Vegetable intake							0.01
≤3 days/week	925	1.0 (ref)		122	1.0 (ref)		
>3 days/week	10,489	0.91 (0.84–0.98)	0.02	1,896	1.20 (0.99–1.46)	0.06	
Fruit intake							0.59
≤3 days/week	6,347	1.0 (ref)		1,080	1.0 (ref)		
>3 days/week	5,067	1.01 (0.96–1.05)	0.72	938	1.03 (0.93–1.13)	0.61	
Red meat intake							0.31
≤3 days/week	7,268	1.0 (ref)		1,246	1.0 (ref)		
>3 days/week	4,146	1.09 (1.04–1.14)	<0.001	772	1.15 (1.04–1.26)	0.01	
Cereal intake							0.40
≤3 days/week	8,308	1.0 (ref)		1,446	1.0 (ref)		
>3 days/week	3,106	0.97 (0.92–1.01)	0.16	572	1.01 (0.91–1.12)	0.82	
Prevalent dyslipidemia							0.58
No	10,515	1.0 (ref)		1,860	1.0 (ref)		
Yes	899	1.13 (1.04–1.23)	0.003	158	1.11 (0.93–1.31)	0.25	
Type 2 diabetes							0.08
No	10,235	1.0 (ref)		1,786	1.0 (ref)		
Yes	1,179	1.16 (1.08–1.24)	<0.001	232	1.31 (1.14–1.51)	<0.001	
Family history of CRC							0.96
No	10,900	1.0 (ref)		1,927	1.0 (ref)		
Yes	514	1.22 (1.10–1.35)	<0.001	91	1.21 (0.97–1.51)	0.08	

BMI, body mass index; CI, confidence interval; CRC, colorectal cancer; OR, odds ratio.

[a] Multivariable logistic analysis included sex, age, BMI, smoking, alcohol consumption, dietary intake of vegetables, fruit, red meat, and cereal, prevalent dyslipidemia and type 2 diabetes, and family history of CRC.

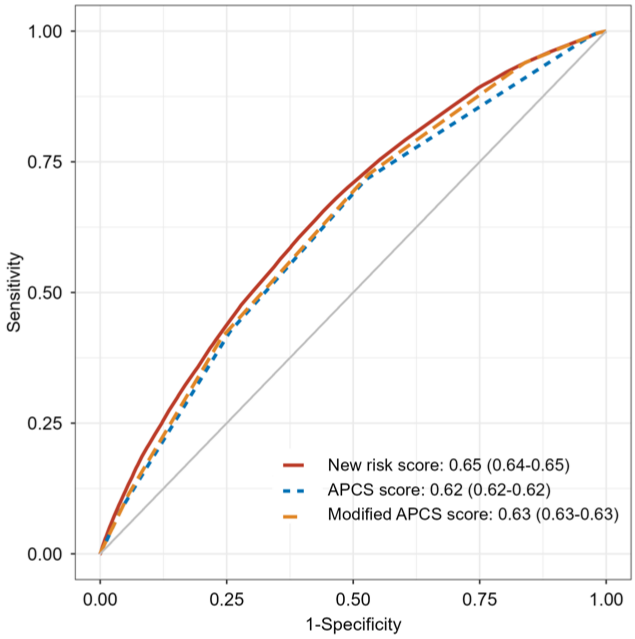

eFigure 1. Receiver operating characteristic curves for colorectal cancer based on the new risk score, Asia-Pacific Colorectal Screening (APCS) score and the modified APCS score
